# Long-term mortality and recurrent vascular events in lacunar versus non-lacunar ischaemic stroke: A cohort study

**DOI:** 10.1177/23969873211062019

**Published:** 2021-12-30

**Authors:** Suzanne Portegijs, Ariel Y Ong, Nynke Halbesma, Aidan Hutchison, Cathie LM Sudlow, Caroline A Jackson

**Affiliations:** 1 Usher Institute, 172239 University of Edinburgh, Edinburgh, Scotland; 2 1190 Vrije Universiteit Amsterdam, Amsterdam, the Netherlands; 3 3129 NHS Lothian, Edinburgh, Scotland; 4 Centre for Clinical Brain Sciences, 3124 University of Edinburgh, Edinburgh, Scotland

**Keywords:** Ischaemic stroke, lacunar stroke, small vessel disease, mortality, recurrent stroke, myocardial infarction

## Abstract

**Introduction:**

Studies of differences in very long-term outcomes between people with lacunar/small vessel disease (SVD) versus other types of ischaemic stroke report mixed findings, with limited data on myocardial infarction (MI). We investigated whether long-term mortality, recurrent stroke and MI risks differ in people with versus without lacunar/SVD ischaemic stroke.

**Patients and methods:**

We included first-ever strokes from a hospital-based stroke cohort study recruited in 2002–2005. We compared risks of death, recurrent stroke and MI during follow-up among lacunar/SVD versus other ischaemic stroke subtypes using Cox regression, adjusting for confounding factors.

**Results:**

We included 812 participants, 283 with lacunar/SVD ischaemic stroke and 529 with other stroke. During a median of 9.2 years (interquartile range 3.1–11.8), there were 519 deaths, 181 recurrent strokes and 79 MIs. Lacunar/SVD stroke was associated with lower mortality (adjusted HR 0.79, 95% CI 0.65 to 0.95), largely due to markedly lower all-cause mortality in the first year. From one year onwards this difference attenuated, with all-cause mortality only slightly and not statistically significantly lower in the lacunar/SVD group (0.86, 95% CI 0.70 to 1.05). There was no clear difference in risk of recurrent stroke (HR 0.84, 95% CI 0.61–1.15) or MI (HR 0.83, 95% CI 0.52–1.34).

**Conclusion:**

Long-term risks of all-cause mortality, recurrent stroke and MI are similar, or only slightly lower, in patients with lacunar/SVD as compared to other ischaemic stroke. Patients and physicians should be as vigilant in optimising short- and long-term secondary prevention of vascular events in lacunar/SVD as for other stroke types.

## Introduction

In high-income settings, ischaemic strokes comprise about 85% of all strokes, around 25% of which are ‘lacunar’ strokes, attributed to small vessel disease (SVD) affecting the deep penetrating arterioles of the brain.^1,2^ Until relatively recently, compared with other stroke subtypes, lacunar/SVD strokes were considered to be relatively benign, due to lower stroke severity at initial presentation and lower early case fatality.^3,4^ However, lacunar/SVD strokes are now recognised to have substantial long-term consequences, including physical and cognitive decline.^3,5^

In a previous systematic review and meta-analysis, we found lower risks of death and recurrent stroke in the first month after lacunar/SVD versus other ischaemic stroke subtypes, with attenuation of these differences thereafter.^6^ However, very few studies reported on long-term outcomes, comparisons of recurrent stroke risk beyond one month were limited by low precision and risk of myocardial infarction (MI) among ischaemic stroke subtypes had rarely been investigated. Our own hospital-based stroke cohort study, with follow-up for up to four years, subsequently reported a lower early risk of recurrent stroke among people with lacunar/SVD versus other ischaemic stroke subtypes, and a trend towards a reduced risk of MI among those with lacunar/SVD stroke (but based on small numbers of MI).^7^ Over the last decade, more studies have reported on outcomes among different ischaemic stroke subtype groups over a longer follow-up period (exceeding five years), but findings appear somewhat mixed and data on risks of MI remain scarce.^8–12^

Differences in long-term prognosis for vascular events following different subtypes of ischaemic stroke may have important implications for clinical management, particularly vascular secondary prevention. In the present study, we extended the follow-up of our hospital-based stroke cohort for up to 14 years through individual-level linkages to Scotland’s national hospital admission and mortality databases, to compare long-term risks of death, recurrent stroke and MI among people with lacunar/SVD versus other ischaemic stroke subtypes.

### Patients and methods

This article is presented in accordance with the Strengthening the Reporting of Observational Studies in Epidemiology (STROBE) statement. The study was approved by the relevant regional Research Ethics Committee (LREC 2001/4/46), and the Scottish National Health Service (NHS) Public Benefit and Privacy Panel provided approval for data linkage.

### Setting and study population

We included participants recruited to the Edinburgh Stroke Study (ESS), a prospective cohort of consecutive consenting adult patients with ischaemic or haemorrhagic stroke (defined according to the classical WHO criteria^13^), admitted as inpatients or referred by primary care physicians to the stroke/transient ischaemic attack outpatient clinic at a large university hospital from 2002–2005. At recruitment, we collected information on stroke onset, symptoms and signs, clinical risk factors, premorbid functional status, lifestyle factors and results of clinical investigations. We obtained informed consent, including for long-term follow-up through linkage to national health records, from all recruited patients, or from relatives (or where necessary through waiver of consent) when patients were unable to provide consent.

For the present study, we linked our original cohort to all Scottish general hospital inpatient records and national death records and included patients with a first-ever ischaemic stroke aged 40 years or more at recruitment.

### Ischaemic stroke subtype classification

**Figure 1.**
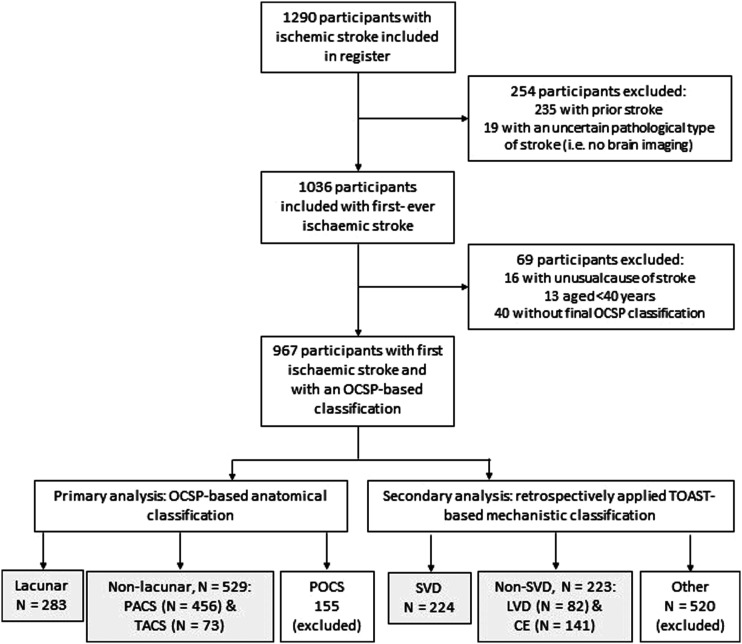
Flow diagram of patients included from the Edinburgh Stroke Study and assigned an anatomical and/or mechanistic ischaemic stroke subtype classification. CE = cardioembolic; LVD = large vessel disease; OCSP = Oxfordshire Community Stroke Project; POCS = posterior circulation stroke; SVD = small vessel disease; TOAST = Trial of Org 10172 in Acute Stroke Treatment.

We categorised ischaemic stroke subtypes according to an anatomical classification based on clinical and brain imaging features. We used the Oxfordshire Community Stroke Project (OCSP) classification to assign a clinical syndrome indicating the presumed site and size of the causative infarct using the clinical features of the stroke which was then modified if necessary by the findings on brain imaging if an infarct considered relevant to the presenting stroke was present (Figure 1; Supplementary Table 1).

To allow comparisons with studies that used a mechanistic ischaemic stroke classification, we retrospectively applied a modified Trial of Org 10172 in Acute Stroke Treatment (TOAST) classification^14^ to create ischaemic stroke subtype groups based on presumed stroke mechanism (Supplementary Table 1). In secondary analyses, we compared SVD versus large vessel disease (LVD) or cardioembolic (CE) stroke.

### Follow-up and outcome definitions

During the original study, we used a ‘hot pursuit’ method to prospectively follow patients for one to four years post-stroke for death, recurrent stroke and MI. These methods included multiple overlapping sources, including alerts from clinical colleagues, patient questionnaires, contact with general practitioners and death certificates. Whenever possible, we arranged for specialist review and investigation of patients with a suspected recurrent stroke. For patients with suspected MI, and those unable to attend a clinical assessment for suspected recurrent stroke, we confirmed or refuted events through review of paper and electronic medical records. In the present study, we ascertained recurrent stroke during the first year post-stroke solely from the prospective hot pursuit methods (defining recurrent stroke using the same WHO definition as for index stroke and requiring a period of neurological stability of 24 hours between index and recurrent stroke, and exclusion of other potential causes of neurological deterioration). Relying on linkages to coded hospital admission data to identify recurrences during this period, when recurrence risk is highest, would have underestimated early recurrence risk. From one-year post-stroke onwards, we identified recurrent strokes from linkage, via a unique community health index number, to hospital admission and mortality records. To optimise the accurate identification of clinically symptomatic strokes fulfilling the WHO definition and occurring beyond one-year of the index stroke, we used stroke-specific ICD-10 codes (i.e. I60, I61, I63 and I64), appearing in the primary or secondary hospital diagnosis or cause of death data fields.^15^

We ascertained MI events during the whole period from hospital admission or mortality records which indicated an acute MI (ICD-10 I21) in the primary or secondary position and from the ‘hot pursuit’ prospective follow-up phase of the ESS (where MI was defined as either autopsy evidence or at least two of the following: symptoms of myocardial ischaemia [e.g. chest pain]; enzyme changes indicative of MI [generally an acute rise in troponin level]; and ECG changes suggesting new ischaemia [new ST-T wave changes, Q waves or left bundle branch block] or sudden death without evidence of an alternative cause).

We identified all-cause mortality through linkage to coded, national mortality records.

### Statistical analysis

We performed statistical analyses using SPSS version 22 (IBM, New York, USA) and Stata version 14. We compared baseline characteristics using the χ^2^ test for categorical variables, Student’s t-test for continuous variables and tests for differences for non-normally distributed variables. For each outcome, we used Kaplan–Meier survival analysis to obtain 1-year, 5-year and 10-year cumulative incidence with 95% confidence intervals (CIs) for lacunar/SVD and other ischaemic subtype groups. We compared people with lacunar/SVD versus other ischaemic strokes, using Cox regression analysis to obtain age- and sex- adjusted and additionally adjusted hazard ratios (HRs) with 95% CIs for each outcome, for the entire follow-up time, 0–1 year and one year to end of follow-up. In the models for mortality and recurrent stroke, we adjusted for age, sex, history of ischaemic heart disease, atrial fibrillation, cardiac failure and smoking status. In the analysis of MI, we adjusted for age, sex, history of ischaemic heart disease, atrial fibrillation and smoking. We followed patients from stroke date to date of outcome, death or end of follow-up (31 December 2015). Information on one or more covariates was missing in only 12 patients and so we performed a complete-case analysis. We checked models for violation of the proportional hazard assumption using log minus log plots and plotting the Schoenfeld residuals.

We performed sensitivity analyses where we calculated sub-distribution HRs for recurrent stroke and MI (treating death as a competing risk). Since stroke severity was not documented for the majority of patients, we repeated the analyses for each outcome stratifying by inpatient/outpatient status at recruitment, as a proxy for more versus less severe strokes. We also performed a sensitivity analysis excluding patients who died within the first three months of the stroke event.

## Results

We included 812 patients with first-ever ischaemic stroke (283 lacunar/SVD, 529 other ischaemic; Figure 1), followed up for a maximum of 14 years (median 9.2, IQR 3.1–11.8). Of these 812 patients, 509 had a visible relevant infarct on their scan, 64 (13%) of whom were allocated to a different comparison group (i.e. lacunar/SVD or other ischaemic stroke type) than would have been the case based on their clinical syndrome alone, with lacunar and non-lacunar strokes equally misclassified. Applying this to the number with no visible infarct, we estimate that 6% of participants were residually misclassified between comparison groups. The inclusion of a slightly higher proportion of lacunar patients (35%) than might be expected reflects the inclusion of (i) milder strokes recruited from an outpatient clinic servicing the whole city and (ii) inpatients from one of three city hospitals receiving acute stroke patients. Patients with lacunar/SVD stroke were slightly (but not significantly) younger than patients with other ischaemic stroke subtypes and more often male and current smokers. Atrial fibrillation, severe ipsilateral carotid stenosis and previous history of ischaemic heart disease were significantly less common in patients with lacunar/SVD versus other ischaemic stroke (Table 1).

**Table 1.** Baseline characteristics of patients with lacunar/SVD and other ischaemic stroke subtypes.

Characteristic	Lacunar/SVD stroke (N = 283) n (%)	Other ischaemic stroke subtypes (N = 529) n (%)	*P*-value^a^
Age at stroke (mean years ± SD)	68.7 (11.9)	72.5 (11.7)	0.46
Male	170 (60.1)	252 (47.6)	0.001
Prior TIA	49 (17.3)	96 (18.2)	0.76
Hypertension^†^	134 (47.3)	277 (52.4)	0.13
Diabetes mellitus^‡^	36 (12.7)	55 (10.4)	0.32
Prior IHD^§^	57 (20.1)	144 (27.2)	0.03
Cardiac failure^¶^	10 (3.5)	35 (6.6)	0.07
Atrial fibrillation^**^	27 (9.6)	107 (20.2)	<0.001
Ipsilateral carotid stenosis^††^	15 (5.3)	95 (18.0)	<0.001
Smoking	108 (38.4)	153 (29.3)	0.008
Alcohol units/week, median (IQR)	4 (0-15)	1 (0-10)	0.2
Independent in ADL before stroke	276 (97.5)	499 (94.5)	0.05
On antiplatelet or anticoagulant at onset	107 (37.8)	237 (44.8)	0.06
CT performed	221 (78.1)	443 (83.7)	0.04
MRI performed	73 (25.8)	116 (21.9)	0.21

^a^P-value for statistical test of difference between SVD/lacunar versus other ischaemic stroke.

^†^Treated hypertension in medical history.

^‡^Diagnosis of or using medication for diabetes mellitus.

^§^MI, angina or coronary revascularisation in medical history.

^¶^Clinical signs of heart failure or taking at least two drugs for its treatment.

^**^History of paroxysmal or persistent atrial fibrillation.

^††^≥70% internal carotid artery stenosis (missing in 11 patients with Lacunar/SVD stroke and 43 patients with other ischaemic stroke subtypes).

^‡‡^Taking any antiplatelet or warfarin at onset of stroke.

ADL - activities of daily living; CT - computed tomography; IHD - ischaemic heart disease; IQR - interquartile range; MRI - magnetic resonance imaging; OCSP - Oxfordshire Community Stroke Project Subtype Classification; TIA - transient ischaemic attack.

**Figure 2.**
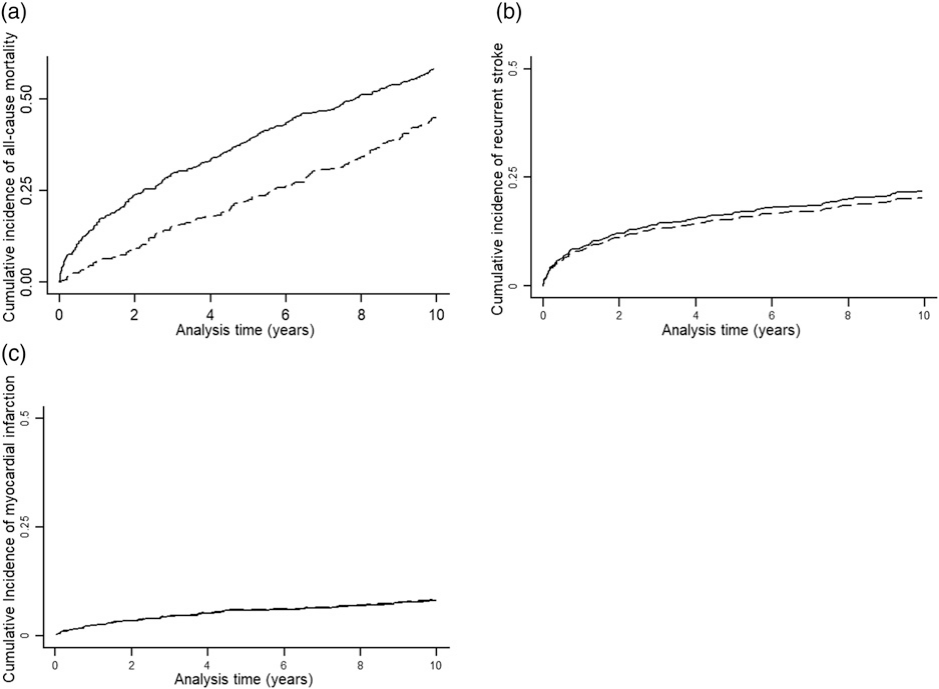
Kaplan–Meier survival graphs showing cumulative incidence of (A) all-cause mortality, (B) recurrent stroke and (C) myocardial infarction, among people with lacunar (dotted line) and other (non-lacunar) ischaemic stroke (solid line).

During follow-up, 159 and 360 deaths occurred among patients with lacunar/SVD and other ischaemic stroke, respectively. During the first year, cumulative mortality was lower among people with lacunar/SVD than other ischaemic stroke. This difference persisted at 5 years and 10 years, but attenuated between 5 and 10 years (Figure 2(a) and Table 2). In adjusted analyses, people with lacunar/SVD stroke had lower mortality risk than other ischaemic stroke subtypes over the entire follow-up period (adjusted HR lacunar/SVD vs others: 0.79, 95% CI 0.65 to 0.95; p = 0.015), largely explained by a much lower risk of dying after lacunar/SVD stroke in the first year (0–1 year HR 0.49, 95% CI 0.28 to 0.84). From one year onwards, the difference in risk attenuated, suggesting only a slightly lower mortality risk among the lacunar/SVD group, although this did not reach statistical significance (0.86, 95% CI 0.70 to 1.05; Table 3). When we excluded patients who died within three months of the stroke, we found that, among 766 remaining patients, those with a lacunar/SVD stroke had an 18% lower mortality risk than other ischaemic stroke (adjusted HR 0.82, 95% CI 0.67 to 1.00, *p* = 0.045).

**Table 2.** Cumulative incidence of mortality, recurrent stroke and myocardial infarction at 1, 5 and 10 years, in patients with lacunar/SVD and other (non-lacunar/non-SVD) ischaemic stroke subtypes.

	Lacunar/SVD (N = 283)		Other ischaemic stroke subtypes (N = 529)	
Outcome	Number of events	Cumulative incidence (95% CI)	Number of events	Cumulative incidence (95% CI)
Mortality				
1 year	16	5.7 (3.5–9.1)	83	15.7 (12.9–19.1)
5 years	63	22.3 (17.9–27.6)	205	38.8 (34.8–43.1)
10 years	128	45.2 (39.7–51.2)	309	58.4 (54.3–62.6)
Recurrent stroke				
1 year	21	7.6 (5.0–11.4)	49	9.9 (12.9–7.6)
5 years	43	16.3 (12.4–21.4)	91	20.1 (16.7–24.2)
10 years	55	22.3 (17.5–28.1)	118	29.2 (24.9–34.2)
Myocardial infarction				
1 year	6	2.2 (1.0–4.8)	16	3.3 (2.0–5.3)
5 years	13	5.0 (2.9–8.5)	40	9.3 (6.9–12.5)
10 years	25	11.1 (7.6–16.1)	47	11.9 (8.9–15.6)

CI - confidence interval; SVD - small vessel disease.

**Table 3.** Hazard ratios from Cox proportional hazard regression analyses for all-cause mortality, recurrent stroke and myocardial infarction, comparing lacunar/SVD versus other (non-lacunar/non-SVD) ischaemic stroke subtypes, by follow-up time period.

Outcome	Entire follow-up period^a^ HR (95% CI)	0–1 year HR (95% CI)	1 year onwards HR (95% CI)
Mortality	(n = 508)	(n = 95)	(n = 413)
Age- and sex- adjusted	0.75 (0.62–0.91)^†^	0.43 (0.25–0.74)^†^	0.83 (0.67–1.01)^‡^
“Fully adjusted”^§^	0.79 (0.65–0.95)^¶^	0.49 (0.28–0.84)^**^	0.86 (0.70–1.05)^**^
Recurrent stroke	(n = 180)	(n = 69)	(n = 111)
Age- and sex- adjusted	0.80 (0.59–1.10)^††^	0.79 (0.47-1.33)^††^	0.81 (0.55–1.20)^††^
“Fully adjusted”^§^	0.84 (0.61–1.15)^††^	0.82 (0.48–1.40)^††^	0.84 (0.57–1.25)^††^
Myocardial infarction	(n = 79)	(n = 22)	(n = 57)
Age- and sex- adjusted	0.82 (0.51–1.31)^††^	0.74 (0.29–1.92)^††^	0.91 (0.53–1.56)^††^
“Fully adjusted”^‡‡^	0.83 (0.52–1.34)^††^	NC	0.93 (0.54–1.60)^††^

^a^800 people with complete information on all covariates included in analyses for the entire time period.

^†^*P*-value 0.002

^‡^*p*-value 0.06.

§ In addition to age and sex, also adjusted for history of ischaemic heart disease, atrial fibrillation, history of cardiac failure and smoking status (all of which were associated with type of stroke and had none or almost no missing values); inclusion of independence in activities of daily living (ADL) did not contribute to the fit of the model and so was not included in the final fully adjusted model.

¶ *P*-value 0.02.

^**^p-value 0.01.

^††^*p*-value >0.05.

^‡‡^Adjusted for age, sex, history of ischaemic heart disease, atrial fibrillation and smoking (not adjusted for history of cardiac failure due to smaller number of outcome events).

CI - confidence interval; HR - hazard ratio; n - number of deaths, recurrent strokes or myocardial infarction events occurring during each time period; NC - not calculated (too few myocardial infarctions within the first year to adjust for additional covariates); SVD - small vessel disease

During the entire follow-up period, 59 and 122 recurrent strokes occurred among patients with lacunar/SVD and other ischaemic stroke, respectively. At 1, 5 and 10 years, there was little difference in the cumulative incidence of recurrent stroke (Figure 2(d) and Table 2). In age- and sex-adjusted and fully adjusted analyses, there was no statistically significant difference in the risk of recurrence between lacunar/SVD versus other ischaemic stroke subtypes over the entire follow-up period (fully adjusted HR: 0.84, 95% CI 0.61 to 1.15), or when restricting the time period to 0–1 year and to one-year onwards (Table 3).

There were 79 MI events (27 and 52 among those with lacunar/SVD and other ischaemic stroke, respectively), with the cumulative incidence of MI similar in both groups at 1, 5 and 10 years (Figure 2(c) and Table 2). In age- and sex-adjusted analyses, there was no statistically significant difference in risk of MI between lacunar/SVD versus other ischaemic stroke (HR 1.0, 95% CI 0.62 to 1.65; Table 3). Similar results were obtained when adjusting for additional confounders and when examining the 0–1 year and 1-year onwards time-periods (Table 3).

We observed similar associations for recurrent stroke and MI risk when we accounted for the competing risk of death. When stratifying by inpatient/outpatient status, results were similar among inpatients as in our primary analysis, but there were no differences in death or recurrent stroke among lacunar/SVD versus other ischaemic stroke patients assessed as outpatients (Supplementary Tables 2 and 3).

In secondary analyses using the mechanistic (TOAST-based) classification, 224 ischaemic strokes were classified as SVD, 82 as LVD and 141 as CE. A large proportion of ischaemic strokes (53.7%) had no determined mechanistic subtype due to having either more than one potential aetiology or undetermined aetiology, and so were excluded (Supplementary Table 4). We obtained similar results to the primary analyses when comparing cumulative incidence of mortality, recurrent stoke and MI at one, five and 10 years among SVD versus non-SVD ischaemic subtypes, although differences were generally more marked between SVD and CE than SVD and LVD stroke. Results of survival analyses were broadly similar to those from primary analyses for each outcome (Supplementary Tables 5 and 6).

## Discussion

During 14 years of follow-up, our study found that, in the long-term, all-cause mortality was only slightly lower in patients with lacunar/SVD stroke compared to other ischaemic stroke subtypes. There was no clear difference in the long-term risk of recurrent stroke or MI, although relatively wide estimates did not preclude lower risks in people with lacunar/SVD strokes.

Our findings on long-term mortality generally align with a number of previous studies with five or more years of follow-up, which found similar or only slightly lower mortality rates between lacunar/SVD versus other ischaemic stroke comparison groups.^8,10,12,16–23^ In contrast to this, one study reported a marked increased mortality risk in people with lacunar/SVD stroke, as compared to other ischaemic stroke,^11^ but this may be due to the inclusion of a selected population with very high rates of prior cardiac comorbidities. We found no significant difference in the very long-term risk of recurrent stroke, although relatively wide CIs did not exclude the possibility of a slightly lower risk in the lacunar/SVD group. This is largely in keeping with other studies^9,10,12,16,20,22,24^, including contemporaneous studies^12,16,22,24^, which similarly found no difference in long-term risk of recurrent stroke risk among people with lacunar/SVD versus other ischaemic stroke. Findings on MI risk across the small number of existing studies that have reported on this are inconsistent. Among just four studies reporting on a total of 270 MI or acute coronary events, two found no difference^10,25^, and two found a lower risk of MI in those with lacunar/SVD stroke.^12,24^ This may have been due to: small numbers of MI events in some studies^10,25^; differences in the composition of the non-lacunar/SVD comparison group; and inclusion of all acute coronary events in some studies.

Our study has various strengths. We included a prospectively recruited cohort of stroke patients which comprised both inpatients and outpatients, resulting in a study population which, in this particular setting, is more representative of the general stroke population than studies based on hospital admitted strokes only. Patients were carefully phenotyped in terms of ischaemic stroke subtype classification using the OCSP classification, which has high inter-rater reliability, is predictive of clinical outcomes and is widely used in clinical trials and epidemiological studies.^26,27^ Recurrent strokes occurring within the first year of stroke are particularly well captured, given our multiple hot pursuit methods and use of advanced brain imaging where possible. The capacity for health record linkage in Scotland facilitated long-term follow-up, resulting in one of the largest long-term follow-up studies of ischaemic stroke subtypes, including for the occurrence of MI which has previously rarely been reported.

Our study does have limitations. Although widely used in clinical trials and epidemiological studies, the OCSP classification does have the potential for misclassification of stroke ischaemic stroke subtypes. Assignment of OCSP based on clinical symptoms alone results in approximately 20 percent of lacunar strokes being misclassified as cortical stroke and vice versa.^28,29^ However, we sought to mitigate this potential for misclassification by using a clinical and imaging-based approach, thus reducing this misclassification. Unfortunately our study was not resourced to perform MRI on all index strokes and so some residual misclassification of ischaemic stroke subtypes is likely.^30-32^ The alternative mechanistic classification method available at the time of patient recruitment was the TOAST classification,^14^ which was limited by concerns around the definition of lacunar stroke and the large proportion of patients with multiple or no determined causes.^33,34^ Whilst new aetiological ischaemic stroke classifications have been developed since, these remain hampered by important limitations, including reliability, validity and ease of application in a clinical setting.^35^ Our study will not have captured recurrent strokes assessed in outpatients beyond the first year of follow-up. Since people with lacunar/SVD strokes may be more likely to have a lacunar/SVD recurrence^7,33^ and lacunar/SVD strokes are more likely than other stroke subtypes to be assessed in an outpatient setting, we may have underestimated the longer term risk of recurrent stroke, particularly in lacunar/SVD stroke patients. Using routinely collected data may also introduce the possibility of diagnostic or recording errors.^36^ Unfortunately we were unable to adjust for stroke severity. However, it is interesting that analysis by inpatient/outpatient assessment status (used as a proxy for severity) found similar findings for inpatients but not outpatients. The requirement for consent meant that not all eligible patients were included. However, as shown previously,^37^ we recruited 88% of eligible patients and found no difference in age, sex and stroke subtype distribution between participants and non-participants. We did find that participants were more likely to be admitted to a stroke unit and were more affluent. Finally, some loss to follow-up may have occurred through migration out of Scotland, but this will be minimal given the age of our cohort and migration occurring mainly among younger people.^38^

The results of our study emphasise the need for clinicians, patients and carers to recognise the non-benign nature of lacunar/SVD stroke, despite symptoms being milder than other types of ischaemic stroke and the importance of adhering to secondary prevention medication. These findings complement the evidence from randomised controlled trials which demonstrates that the relative effect of statins and blood pressure lowering and antiplatelet medications over the long-term does not differ between ischaemic stroke subtypes.^39^ Our findings that lacunar ischaemic stroke patients have a similar prognosis for relevant outcomes as other ischaemic stroke patients, and therefore as much to gain through long-term preventive efforts, help to emphasise the importance of such preventive efforts in all ischaemic stroke patients, as per clinical guidelines. Under ideal circumstances, secondary stroke prevention treatment is thought to reduce risk of recurrent stroke by about 80%.^40^ However, secondary prevention has been shown to be sub-optimal, through failure to successfully translate evidence-based recommendations into clinical practice, particularly in low-income countries.^40^ Non-adherence to prescribed secondary prevention medication increases with time from stroke and is associated with low perceived benefit of medication and younger age.^40,41^

Further research, including pooling of relevant studies to improve study power and harmonise key definitions, is needed to conclusively establish whether MI risk differs between lacunar/SVD versus other ischaemic stroke subtypes. We should also identify reasons for sub-optimal implementation of clinical guidelines on secondary prevention and determine whether adherence differs between those with lacunar versus other stroke subtypes.

## Conclusion

In the long-term, patients with lacunar/SVD stroke may have only a slightly lower risk of death compared to patients with other ischaemic strokes. There is no conclusive evidence that recurrent stroke and MI risks differ between these groups. Patients and physicians should be as vigilant in optimising short and long-term secondary prevention of vascular events among patients following lacunar/SVD stroke as for other types of ischaemic stroke.

## Supplemental Material

sj-pdf-1-eso-10.1177_23969873211062019 – Supplemental Material for Long-term mortality and recurrent vascular events in lacunar versus non-lacunar ischaemic stroke: A cohort study Click here for additional data file. Supplemental Material, sj-pdf-1-eso-10.1177_23969873211062019 for Long-term mortality and recurrent vascular events in lacunar versus non-lacunar ischaemic stroke: A cohort study by S Portegijs, AY Ong, N Halbesma, A Hutchinson, Sudlow CLM, and CA Jackson in European Stroke Journal
