# Supplementary material for: Long-term mortality and recurrent vascular events in lacunar versus non-lacunar ischaemic stroke: A cohort study
Source: Eur Stroke J. 2021 Dec 30;7(1):57–65. doi: 10.1177/23969873211062019 (PMC8915237; doi:10.1177/23969873211062019)
Supplement: sj-pdf-1-eso-10.1177_23969873211062019 – Supplemental Material for Long-term mortality and recurrent vascular events in lacunar versus non-lacunar ischaemic stroke: A cohort study [file sj-pdf-1-eso-10.1177_23969873211062019.pdf]

Long-term mortality and recurrent vascular events in lacunar versus non-lacunar ischemic stroke: a cohort study

Portegijs S (MSc)[1,2*], Ong AY (MBChB)[3*], Halbesma N (PhD)[1], Hutchinson A (MSc)[1,4], Sudlow CLM (DPhil)[1,4] Jackson CA (PhD)[1†]

Supplementary material

Supplementary Table 1 Description of the OCSP-based anatomical classification and the TOAST-based mechanistic classification methods

Category	Description
OCSP-based anatomical classification	
SVD/lacunar infarction (LACI)	i. Clinical presentation with any of:<br>- Pure motor stroke<br>- Pure sensory stroke<br>- Sensorimotor stroke<br>(with the above deficit involving at least two contiguous areas out of three of the whole of the face, arm and leg)<br>- Ataxic hemiparesis<br>AND<br>Either no visible relevant infarct or a visible relevant subcortical infarct (located in the thalamus, basal ganglia, internal or external capsule or centrum semiovale) measuring ≤ 20mm on brain imaging)<br><br>ii. A cortical syndrome with a relevant subcortical infarct that accounted for their symptoms
Non-lacunar (partial anterior circulation infarction [PACI] or total anterior circulation infarction [TACI])	▪ Presentation with a cortical-involving anterior circulation syndrome with or without an accompanying visible relevant cortical or striatocapsular infarct on brain imaging<br>OR<br>▪ Presentation with a clinical lacunar or posterior circulation syndrome but with a cortical-involving or striatocapsular infarct in the anterior circulation territory that was clearly relevant to the presenting stroke
Posterior circulation infarction (POCI)	▪ Presentation with a posterior circulation syndrome with or without an accompanying relevant infarct on brain imaging, or with an anterior or cortical syndrome but a visible relevant infarct in the posterior circulation
Modified TOAST-based mechanistic classification	
Small vessel disease	▪ Clinical findings of one of the lacunar syndromes should be present. Brain imaging should be normal or show a relevant brain stem or subcortical hemispheric lesion of diameter <1.5cm<br>▪ A history of diabetes mellitus or hypertension supports the diagnosis<br>▪ Potential cardiac sources of embolism, such as AF, should be absent, and the large extracranial arteries should not demonstrate ≥70% stenosis*
Large vessel disease	▪ Clinical findings include cortical, cerebellar, or brain stem dysfunction and on brain imaging cortical, cerebellar, brain stem or subcortical lesions >1.5cm are considered to be of potential large artery atherosclerotic origin<br>▪ Diagnosis requires supportive evidence by duplex imaging or arteriography of ≥70%* stenosis of an appropriate intracranial or extracranial artery<br>▪ Potential sources of cardiogenic embolism, such as AF should be excluded, and history of TIAs in the same vascular territory

	supports the clinical diagnosis
Cardioembolism	▪ Clinical and brain imaging findings are similar to those described for large artery atherosclerosis<br>▪ At least 1 cardiac source of embolism, such as AF, must be identified. Previous TIAs in >1 vascular territory supports the diagnosis<br>▪ Potential large artery atherosclerotic sources of thrombosis or embolism should be absent
'Other' aetiology	
I. Multiple aetiologies	Includes patients with ≥2 potential causes of stroke (e.g. AF and >70% stenosis of extracranial arteries)
II. Undetermined aetiology with complete investigation	Includes patients with no identifiable cause of stroke following complete investigation
III. Undetermined aetiology with incomplete investigation	Includes patients with no identifiable cause of stroke following incomplete investigation

*Using the European Carotid Surgery scale for measuring stenosis, which is equivalent to 50% on the North American Symptomatic Carotid Endarterectomy Trial scale
AF = atrial fibrillation; OCSP = Oxfordshire Community Stroke Project; TIA = transient ischaemic attack; TOAST = Trial of Org 10172 in Acute Stroke Treatment

Supplementary Table 2 Subdistribution hazard ratios from Cox proportional hazard regression analyses, accounting for competing risk of death, for recurrent stroke and MI, comparing lacunar/SVD vs other ischaemic stroke subtypes (as defined by OCSP-based anatomical classification), by time period

	**Time period**		
**Outcome**	**Entire follow-up**	**0-1 year**	**1 year onwards**
Recurrent stroke			
Age- and sex-adjusted	0.92 (0.67 to 1.26)	0.87 (0.52 to 1.5)	0.88 (0.59 to 1.3)
Fully adjusted*	0.93 (0.67 to 1.26)	0.84 (0.50 to 1.4)	0.9 (0.60 to 1.3)
Myocardial infarction			
Age- and sex-adjusted	0.99 (0.61 to 1.61)	0.74 (0.27 to 2.01)	1.00 (0.58 to 1.74)
Fully adjusted*	0.95 (0.58 to 1.56)	NC	0.95 (0.54 to 1.66)†

*Adjusted for age, sex, prior ischaemic heart disease, atrial fibrillation, prior cardiac failure and smoking
†Due to smaller number of events, adjusted for age, sex, prior ischaemic heart disease, atrial fibrillation and smoking (and not prior cardiac failure)
NC = not calculated (too few events to adjust for covariates other than age and sex)

Supplementary Table 3 Hazard ratios from Cox proportional hazard regression analyses for OCSP-based anatomical classification for all-cause mortality and recurrent stroke, split by different time periods and stratified by inpatient and outpatient status, comparing lacunar/SVD vs other ischaemic stroke subtypes

	**Inpatients (N = 363)**			**Outpatients (N = 449)**		
**Outcome**	**Entire follow-up**	**Year**	**1-year onward**	**Entire follow-up**	**year**	**1 year onwards**
Mortality	(n= 266)	(n = 83)	(n = 183)	(n = 253)	(n = 17)	(n = 256)
Age-and sex-adjusted	0.60 (0.45-0.80)	0.40 (0.22-0.74)	0.69 (0.50-0.95)	0.93 (0.72-1.20)	0.94 (0.73-1.21)	0.94 (0.72-1.23)
Fully adjusted*	0.67 (0.50-0.90)	0.44 (0.23-0.85)	0.75 (0.54-1.04)	0.92 (0.71-1.20)	NC	0.93 (0.72-1.22)
Recurrent stroke	(n = 86)	(n = 36)	(n = 50)	(n = 95)	(n = 32)	(n = 63)
Age-and sex-adjusted	0.74 (0.47-1.17)	0.85 (0.42-1.73)	0.67 (0.37-1.22)	0.76 (0.49-1.18)	0.68 (0.32-1.45)	0.84 (0.49-1.45)
Fully adjusted*	0.78 (0.48-1.26)	NC	0.65 (0.35-1.22)	0.76 (0.48-1.19)	NC	0.78 (0.44-1.36)

*Adjusted for age, sex, smoking, history of ischaemic heart disease, history of heart failure and atrial fibrillation
NC = not calculated (given too few outcomes)

Supplementary Table 4 Baseline characteristics of participants, by ischaemic subtype category defined according to mechanistic TOAST-based classification

Characteristic	SVD (N=224) n (%)	Non-SVD[*] (N=223) n (%)	LVD (N=82) n (%)	CE (N=141) n (%)	Other[†] (N=520) n (%)
Age at stroke (mean ± SD)	67.8 (12.0)	74.2 (10.7)	70.6 (10.3)	76.3(10.4)	71.2 (11.7)
Male	135 (60.3)	113 (50.7)	45 (54.9)	68 (48.2)	250 (48.1)
Prior TIA	37 (16.5)	37 (16.7)	17 (20.7)	20 (14.3)	91 (17.5)
Hypertension[‡]	107 (47.8)	121 (54.5)	46 (56.1)	75 (53.6)	256 (49.2)
Diabetes mellitus[§]	29 (12.9)	28 (12.6)	12 (14.6)	16 (11.3)	54 (10.4)
Prior IHD[¶]	41 (18.3)	69 (30.9)	16 (19.5)	53 (37.6)	125 (24.0)
Cardiac failure[**]	5 (2.2)	23 (10.4)	3 (3.7)	20 (14.3)	26 (5.0)
Atrial fibrillation[††]	0 (0)	91 (40.8)	0 (0)	91 (64.5)	64 (12.3)
Ipsilateral carotid Stenosis[‡‡]	-	82 (36.8)	82 (100)	-	30 (5.8)
Smoking	90 (40.4)	62 (28.2)	36 (45.0)	26 (18.6)	146 (28.5)
Alcohol units/week, median (IQR)	4 (0-15)	1 (0-8)	2 (0-10)	1 (0-8)	1 (0-10)
Independent in ADL before Stroke	220 (98.2)	213 (95.5)	78 (95.1)	135 (95.7)	491 (94.6)
CT	175 (78.1)	187 (83.9)	73 (89.0)	114 (80.9)	421 (81.0)
MRI	56 (25.0)	48 (21.6)	13 (16.0)	35 (24.8)	136 (26.2)

[*]Non-SVD = LVD plus CE strokes
[†]'Other' includes strokes: with multiple aetiologies; undetermined aetiology despite complete investigation and undetermined aetiology with incomplete investigation
[‡]Treated hypertension in medical history
[§]Diagnosis of or using medication for diabetes mellitus
[¶]MI, angina or coronary revascularization in medical history
[**]Clinical signs of heart failure or taking at least two drugs for its treatment
[††]History of or post stroke electrocardiogram evidence of paroxysmal or persistent atrial fibrillation
[‡‡]≥70% internal carotid artery stenosis, based on the European Carotid Surgery scale for measuring stenosis, which is equivalent to 50% on the North American Symptomatic Carotid Endarterectomy Trial scale (there are no values in the SVD or CE columns since ipsilateral carotid stenosis is incorporated into the definition of these subtypes)
ADL = activities of daily living; CE = cardioembolic; CT = computed tomography; IHD = ischaemic heart disease; IQR: = interquartile range; LVD = large vessel disease; MRI = magnetic resonance imaging; SVD = small vessel disease; TIA = transient ischaemic attack

Supplementary Table 5 Cumulative incidence of mortality, recurrent stroke and myocardial infarction by ischaemic stroke subtype, defined according to mechanistic TOAST-based classification

	Small vessel disease (N = 224)		Large vessel disease (N = 82)		Cardioembolic (N =141)	
Outcome	Number of events	Cumulative incidence (95% CI)	Number of events	Cumulative incidence (95% CI)	Number of events	Cumulative incidence (95% CI)
Mortality						
1 year	8	3.6 (1.8 to 7.0)	10	12.2 (6.8 to 21.5)	29	20.6 (14.8 to 28.2)
5 years	47	21.0 (16.2 to 26.9)	33	40.2 (30.6 to 51.7)	73	51.8 (43.8 to 60.2)
10 years	94	42.0 (35.8 to 48.7)	53	64.6 (54.4 to 74.8)	101	71.6 (64.1 to 78.8
Recurrent stroke						
1 year	15	6.8 (4.2 to 11.0)	16	11.5 (6.2 to 21.0)	16	12.3 (7.7 to 19.3)
5 years	34	16.1 (11.8 to 21.8)	17	22.7 (14.4 to 34.6)	31	27.7 (20.1 to 37.2)
10 years	43	21.5 (16.4 to 28.0)	18	25.0 (16.1 to 37.6)	38	38.4 (29.0 to 49.6)
Myocardial infarction						
1 year	2	0.9 (0.2 to 3.6)	1	1.3 (0.2 to 8.9)	6	4.7 (2.1 to 10.2)
5 years	9	4.4 (2.3 to 8.4)	5	7.8 (3.3 to 17.8)	10	9.2 (5.0 to 16.7)
10 years	18	10.2 (6.5 to 15.7)	7	12.1 (5.9 to 24.1)	12	12.5 (7.1 to 21.5)

CI = confidence interval; TOAST = Trial of Org 10172 in Acute Stroke Treatment

Supplementary Table 6 Hazard ratios for risk of all-cause mortality, recurrent stroke and myocardial infarction, comparing stroke attributed to SVD versus non-SVD (i.e. LVD or CE), by time period

	HR (95% CI)		
**Outcome**	**Entire follow-up***	**0-1 year**	**1 year onwards**
Mortality			
Age- and sex-adjusted	0.60 (0.47 to 0.76)†	0.25 (0.11 to 0.53)†	0.68 (0.52 to 0.87)‡
Fully adjusted§	0.62 (0.47 to 0.81)¶	0.27 (0.12 to 0.61)**	0.69 (0.52 to 0.93)††
Recurrent stroke			
Age- and sex-adjusted	0.71 (0.48 to 1.05)	0.62 (0.32 to 1.20)	0.76 (0.46 to 1.26)
Fully adjusted§	0.72 (0.46 to 1.13)	0.54 (0.27 to 1.08)	0.88 (0.48 to 1.61)
Myocardial infarction			
Age- and sex-adjusted	0.72 (0.39 to 1.33)	0.25 (0.05 to 1.31)	0.89 (0.45 to 1.78)
Fully adjusted¶¶	0.72 (0.36 to 1.44)	NC	NC

*442 cases included in analyses of entire follow-up period
†p-value <0.001
‡p-value 0.003
§Analyses of entire follow-up period and 1 year onwards adjusted for age, sex, atrial fibrillation, prior ischaemic heart disease, prior cardiac failure and smoking; analyses for 0-1 year period adjusted for age, sex, atrial fibrillation and prior ischaemic heart disease (unable to incorporate additional covariates due to small number of outcomes)
¶P-value 0.001
**p-value 0.002
††p-value 0.02
¶¶Adjusted for age, sex, atrial fibrillation and history of ischaemic heart disease (unable to incorporate additional covariates due to small number of outcomes)
HR = hazard ratio; CI: confidence interval; HR: NC = not calculated (too few outcomes to adjusted for additional covariates)
